# Association of Tree Nut Consumption with Cardiovascular Disease and Cardiometabolic Risk Factors and Health Outcomes in US Adults: NHANES 2011–2018

**DOI:** 10.1016/j.cdnut.2023.102007

**Published:** 2023-09-25

**Authors:** Stephanie M. Lopez-Neyman, Namvar Zohoori, K. Shane Broughton, Derek C. Miketinas

**Affiliations:** 1 Department of Nutrition Sciences, Texas Woman’s University, Denton, TX, United States; 2 Department of Nutrition Sciences, Texas Woman’s University, Houston, TX, United States; 3 Department of Epidemiology, Fay W. Boozman College of Public Health, University of Arkansas for Medical Sciences, and Arkansas Department of Health, Little Rock, AR, United States

**Keywords:** tree nuts, usual intake, adults, NHANES, risk factors, cardiovascular disease, cardiometabolic, health outcomes, NCI method, nutritional epidemiology

## Abstract

**Background:**

Tree nuts are nutrient dense, and their consumption has been associated with improvements in health outcomes.

**Objective:**

To estimate the usual tree nut intake and examine the association between tree nut consumption and cardiometabolic (CM) health outcomes in a nationally representative sample of US adults.

**Methods:**

Cross-sectional data were analyzed from a sample of 18,150 adults aged ≥ 20y who provided at least one reliable 24-h dietary recall and had complete data for the variables of interest in the NHANES 2011–2018. Tree nut consumers were defined as those consuming ≥ ¼ ounce/d (7.09 g). The National Cancer Institute Method was used to estimate the usual tree nut intake among consumers. Measurement error calibrated regression models were used to assess the association between tree nut consumption and each health outcome of interest.

**Results:**

Approximately 8% of all participants (*n* = 1238) consumed tree nuts and had a mean ± SE usual intake of 39.5 ± 1.8 g/d. Tree nut consumers were less likely to have obesity (31% vs. 40%, *P* < 0.001) and low high-density lipoprotein cholesterol (22% vs. 30%, *P* < 0.001*)* compared with nonconsumers. Moreover, tree nut consumers had a lower mean waist circumference (WC) (97.1 ± 0.7 vs. 100.5 ± 0.3 cm, *P* < 0.001) and apolipoprotein B (87.5 ± 1.2 vs. 91.8 ± 0.5 mg/dL, *P =* 0.004) than nonconsumers. After adjusting models for demographics and lifestyle covariates, the difference in WC between average intake (33.7 g/d) and low threshold intake (7.09/g) of tree nuts was -1.42 ± 0.58 cm (*P* = 0.005).

**Conclusions:**

Most US adults do not consume tree nuts, yet modest consumption was associated with decreased prevalence of cardiovascular disease and CM risk factors and improvement for some health outcome measures.

## Introduction

Cardiovascular disease (CVD) is the leading cause of death worldwide, accounting for 32% of all global deaths [1]. After hypertension, dietary risks are the leading modifiable CVD risk factor, accounting for 6.58 million cardiovascular deaths across 21 global regions in 2021 (2). Nuts and seeds, fruits, vegetables, legumes, and whole grains, were food types included in the dietary risk estimates that were underconsumed globally [2].

Healthy, cardioprotective dietary patterns include plant-based foods such as nuts and seeds [3]. Nuts are rich in unsaturated fatty acids and contain plant protein, fiber, minerals, and phytochemicals such as phytosterols and polyphenols [4,5]. Systematic reviews and meta-analyses of epidemiological and intervention studies demonstrate that frequent nut consumption can improve body mass index (BMI) [6,7], fasting plasma glucose (FPG) [7,8], blood lipid profiles [7,9], and systolic blood pressure (SBP) [7,10]. Moreover, nut consumption is associated with lower risk of all-cause, CVD, and coronary artery disease mortality [11,12].

Cross-sectional analyses of nut consumption were associated with improved CM risk factors in adults across countries [[13], [14], [15], [16], [17], [18]]. Croatian adults reporting weekly and monthly nut intake were less likely to have reduced high-density lipoprotein cholesterol (HDL-C) compared with nonconsumers [13]. Lower blood pressure and risk of hypertension were associated with frequent nut consumption among Iranian adults [14]. Body weight, BMI, and measures of central adiposity were significantly lower for whole and total nut consumers than non-nut consumers in the 2008–2009 New Zealand Adult Nutrition Survey [15]. Among the adult population in the United Kingdom, tree nut snack consumers had lower BMI, waist circumference (WC), SBP, and diastolic blood pressure (DBP), and higher HDL-C than nonconsumers [16]. US adults in the 1999–2004 NHANES who consumed ≥ ¼ ounce of nuts per day had lower BMI, WC, and SBP measures than adults who consumed < ¼ ounce of nuts per day [17]. Better BMI, WC, Homeostatic Model Assessment for Insulin Resistance (HOMA-IR), and a lower likelihood of obesity were associated with tree nut consumption among US adults participating in the NHANES 2005–2010 [18].

O’Neil and colleagues reported the estimates of tree nut consumption using a nationally representative sample of US adults using NHANES 1999–2004 [19] and NHANES 2005–2010 data [20]. Given that frequent nut consumption has a beneficial effect on CVD risk [5,21] there are no recent estimates examining the US population-level intake of tree nuts and their association with CVD and CM risk factors, as well as health outcomes, with the most current NHANES data. Moreover, apolipoprotein B (ApoB) is considered a more accurate cardiovascular disease risk marker than low-density lipoprotein cholesterol (LDL-C) alone [22]. This biomarker has yet to be examined as a CVD risk factor among tree nut consumers using NHANES data [17,18]. Therefore, this secondary data analysis of cross-sectional data [1] determined the usual tree nut intake, [2] examined CVD and CM risk factors by tree nut consumption, [3] and examined the association between tree nut consumption and health outcomes in adults who participated in the NHANES 2011–2018.

## Methods

### Study design and study participants

The NHANES uses a complex, stratified, multistage probability sampling design and is representative of the noninstitutionalized US civilian population. Each NHANES cycle includes data from standardized interviews, physical examinations, and laboratory tests by trained personnel. A detailed description of the survey design, protocol, and methods is published elsewhere [23]. The NCHS Ethics Review Board reviewed and approved the NHANES survey protocol and collected informed consent from study participants [24]. The researcher’s institutional review board determined that this secondary data analysis was exempt from review. The procedures followed were in accordance with the ethical standards of the institution or regional committee on human experimentation and that approval was obtained from the relevant committee on human subjects and/or animal welfare and followed the STROBE- nutritional epidemiology guidelines for cross-sectional studies [25].

**FIGURE 1:**
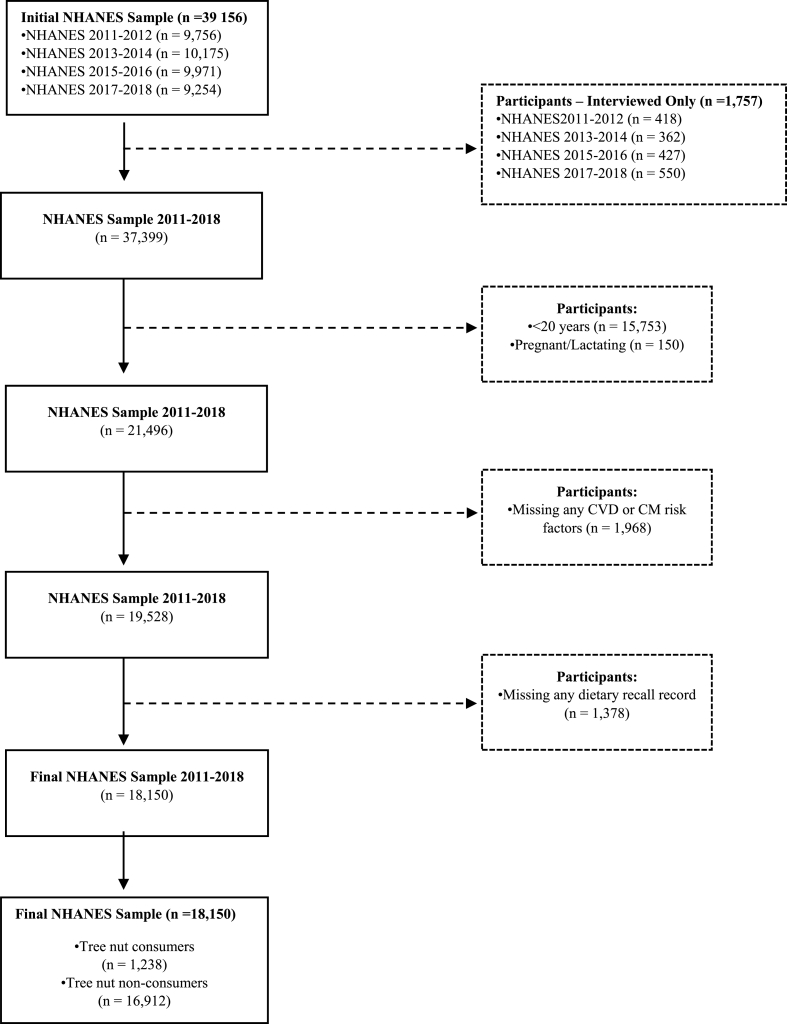
Flow chart of participant selection. CM, cardiometabolic; CVD, cardiovascular disease.

This study analyzed data from 2011–2012, 2013–2014, 2015–2016, and 2017–2018 NHANES (*N* = 39,156 participants). The analyses included nonpregnant, nonlactating adults aged ≥ 20y who provided complete data for all outcomes of interest and at least one reliable 24-h dietary recall, yielding a final study sample size of 18,150 participants (Figure 1).

Demographic and socioeconomic data were obtained from the inperson home interviews by trained interviewers and included age, gender, selfreported race and ethnicity, education level, and poverty income ratio. Age was categorized into 3 groups (20 to 39 y, 40 to 59 y, and ≥ 60 y). Educational level was categorized into less than high school, high school diploma/general equivalency degree, and college or above. The poverty income ratio (PIR) was categorized into < 1.3 (low income), 1.3 to < 3.5 (middle income), and ≥ 3.5 (high income) [26].

### Tree nut consumption and definition

Tree nut consumption was derived from at least one reliable 24-h dietary recall per participant. The dietary recalls were administered by trained dietary interviewers using the Automated Multiple-Pass Method [27] during the mobile center examination. A second dietary recall was collected by telephone 3 to 10 d after the mobile center examination. A detailed description of the dietary recalls and data collection is published elsewhere [28]. The USDA maintains the Food and Nutrient Database for Dietary Studies (FNDDS) and assigns 8-digit food codes to foods and beverages consumed in What We Eat in America, NHANES [29]. We used the 8-digit food codes from the FNDDS 2017–2018 database to identify food ingredients from the study participants' dietary recalls that included all tree nuts (solely as nuts and not part of products): almonds, Brazil nuts, cashews, hazelnuts, macadamias, pecans, pinenuts, pistachios, and walnuts, and determined the participants' day one and day 2 intakes of tree nuts in (g/d). Although peanuts have a similar nutrient profile as tree nuts [5], have beneficial health effects [30], and are widely consumed among Americans [31], they are usually classified as legumes and excluded from many of the previous similar analyses. In addition, meta-analysis of clinical trials [32,33] examining health risk factors of interest for our study excluded peanuts. Consistent with O’Neil and colleagues [19], we defined tree nut consumers as those reporting ≥¼ ounce (7.09 g) per day and nonconsumers as reporting <¼ ounce tree nuts on at least one consumption day—this amount was chosen by O’Neil and colleagues to exclude individuals who consumed small amounts of nuts from nut-containing food products such as cookies, breads, and candies, using a reported per capita tree nut consumption of approximately 7.3 g/d [19,34]. For the regression models, we defined average intake as 33.7 g/d, which was the median intake among tree nut consumers, and we defined low threshold intake as 7.09 [19]. The difference between 33.7 g/d and 7.09 g/d (26.6 g/d) is close to a typical serving size of nuts (1 ounce = 28 g).

### Measures

During the mobile examination center visit, trained technicians used standardized procedures and equipment to collect participants’ anthropometric, physical examination, and laboratory values [28]. Height (cm), weight (kg), and WC(cm) were obtained following NHANES protocols [28]. Measured body weight and height were used to calculate BMI (kg/m^2^). SBP and DBP were measured multiple times during the physical examination, using an average of 3 measurements. Laboratory values were obtained at the mobile examination center using standardized protocols [28].

Nonfasting total cholesterol (TC), nonfasting HDL-C, and fasting TG were measured using a Roche/Hitachi Modular P Chemistry Analyzer (NHANES 2011–2012) and Roche/Hitachi Cobas 6000 Analyzer (NHANES 2013–2018). FPG was measured using Roche/Hitachi Modular P Chemistry Analyzer (NHANES 2011–2012) and Roche/Hitachi Cobas C Chemistry Analyzer (NHANES 2013–2018). Nonfasting HbA1c was measured using Tosh G8 Glycohemoglobin Analyzer (2011–2018). Fasting ApoB was measured using Seimans ProSpec Analyzer (NHANES 2011–2014) and Roche/Hitachi Cobas 6000 Analyzer (NHANES 2015–2016) [28]. Detailed blood collection procedures for laboratory analysis have been described elsewhere [[35], [36], [37], [38]].

Risk factors were dichotomous variables (1 = present, 0 = absent) defined accordingly: obesity, as BMI ≥ 30 kg/m^2^; abdominal obesity, as WC >102 cm in males and >88 cm in females; hypertension, as mean SBP ≥ 130 mmHg, mean DBP ≥80 mmHg, or antihypertensive medication use; elevated TC, as TC ≥ 240 mg/dL, or lipid-lowering medication use; diabetes, as an FPG ≥ 126 mg/dL, HbA1c (≥ 6.5%), or hypoglycemic medication use; elevated ApoB (≥ 100 mg/dL)[39]; reduced HDL-C, as HDL-C < 40 mg/dL (males) and < 50 mg/dL (females) (in the context of metabolic syndrome, low HDL-C cut points differ for males and females [40]; we elected not to use the HDL-C cut point < 40 mg/dL for both males and females) [39]; elevated TG, as TG ≥ 150 mg/dL; and elevated glucose, as FPG ≥ 100 mg/dL or hyperglycemic medication use.

### Statistical analyses

All data analyses were performed using Statistical Analysis Software (SAS) version 9.4 (SAS Institute). Appropriate sample weights were used to account for the NHANES complex sampling design, and significance was considered as *P* < 0.05. Participants’ characteristics were described according to tree nut intake (tree nut consumers compared with nonconsumers) and expressed as percentages ± SE for categorical variables and as means ± SE for continuous variables.

Usual tree nut intake was determined using the National Cancer Institute (NCI) method [41] and the Simulating Intake of Micronutrients for Policy Learning and Engagement (SIMPLE) macro programming wrapper [42]**.** Briefly, the NCI method accounts for within-person variation intake and estimates the usual intake of a food or nutrient using a 2-part model: the probability to consume a food or nutrient multiplied by the amount of the food or nutrient consumed on a consumption day. For ubiquitously consumed foods or nutrients, the probability of consumption is equal to 1, known as the amount-only model [41]. This study used the amount-only model for estimating the usual tree nut intake for tree nut consumers. Details of the NCI method, including the MIXTRAN and DISTRIB macros [41,43], and the SIMPLE macro [42] have been published elsewhere. Covariates used to assess the usual intake include dietary recall sequence, weekend compared with weekday, and race and ethnicity.

Using the NCI method 2-part model, calibrated regression models were used to examine the relationship between the usual tree nut intake and health outcomes (continuous and categorical). The resulting regression models were used to estimate the difference in health outcomes between average intake (33.7 g/d) and low threshold intake (7.09 g/d) of tree nuts. It is important to note that the NCI method does not allow for direct estimation of usual intake for individuals but rather the characteristics of a group [44].

Measurement error calibrated linear and logistic regression models were used to model continuous and dichotomous outcomes, respectively. Two models (Model 1 and Model 2) were fit for each health outcome of interest to test the impact of covariates on the model. Model 1 covariates included age, gender, education, race and ethnicity, PIR, and health insurance status. Model 2 covariates included Model 1 covariates, physical activity, and smoking status. We based our selected covariates for our analytic models on previous studies [17,18], keeping in practice with selecting covariates a priori [45].

## Results

### Demographics and Tree Nut Consumption

The analytical sample included 18,150 adults; ∼8% of the sample (*n* = 1,238) were considered tree nut consumers, representing approximately 16.7 million adults (weighted number). Tree nut consumers were more likely to be female, nonHispanic white, middle-aged and older, of higher income, more educated, and more likely to have insurance coverage (Table 1). Of all participants included in the analysis, 87.3% provided more than one 24-h recall.

**TABLE 1:** Participant characteristics for US adults by tree nut consumption who participated in the National Health and Nutrition Examination Survey, 2011–2018

Characteristics	Tree Nut Consumers	NonConsumers
	≥ 7.09 g/d	< 7.09 g/d
	(*n* = 1238)	(*n* = 16912)
Gender, %		
Female	59.9 ± 1.9	50.2 ± 0.5
Age, y	51.8 ± 0.7	47.7 ± 0.3
Age group, %		
20 to 39y	25.8 ± 2.1	36.0 ± 0.9
40 to 59y	37.4 ± 2.3	36.8 ± 0.6
≥ 60y	36.8 ± 2.2	27.2 ± 0.7
Race/ethnicity, %^1^		
Mexican American	5.7 ± 1.0	8.6 ± 0.9
Other Hispanic	3.7 ± 0.6	6.4 ± 0.6
NonHispanic White	75.3 ± 2.1	65.7 ± 1.7
NonHispanic Black	5.9 ± 0.8	11.1 ± 1.0
NonHispanic Asian	6.0 ± 0.8	4.7 ± 0.2
Education level, %		
< High School	4.7 ± 0.6	14.3 ± 0.7
HSD/GED or equivalent	13.2 ± 1.4	23.6 ± 0.7
≥ College	82.0 ± 1.7	62.1 ± 1.1
Poverty income ratio, %^2^		
< 1.3	11.9 ± 1.2	22.3 ± 0.9
1.3 to <3.5	27.7 ± 2.1	36.3 ± 0.8
≥3.5	60.4 ± 2.6	41.3 ± 1.3
Health insurance, %		
Yes	92.5 ± 0.9	83.3 ± 0.8

GED, general equivalency diploma; HSD, high school diploma, SE, standard error.

Values are weighted means ± SE for continuous variables or weighted percentages ± SE for categorical variables.

Differences between tree nut consumers and non-tree nut consumers, *P* <.001 for all comparisons.

1 Race/ethnicity groups do not sum to 100% because the "other" category is not presented per National Center for Health Statistics analytical guidelines.

2 Poverty income ratio: <1.3 (low income), 1.3 to <3.5 (middle income), ≥3.5 (high income).

Mean usual tree nut intake for tree nut consumers was 39.5 ± 1.8 g/d. Usual intake for those who consumed tree nuts at the 75^th^ percentile was 49.4 ± 2.5 g/d, the median was 33.7 ± 1.7 g/d, and the 25^th^ percentile was 23.0 ± 1.9 g/d. Across race and ethnicity, nonHispanic Blacks (48.2 ± 3.1 g/d) mean usual intake was greater than nonHispanic whites (39.1 ± 2.2 g/d), nonHispanic Asians (35.9 ± 2.5 g/d), Mexican Americans (33.6 ± 4.4 g/d), and other Hispanics (32.5 ± 5.2g/d) as shown in Supplemental Table 1. Mean usual intake for dietary fiber and energy for tree nut consumers was 24.4 ± 0.5 g/d and 2,375 ± 28.7 kcal/d, respectively. Mean usual intake for dietary fiber and energy for nonconsumers was 17.02 ± 0.2 g/d and 2,164 ± 8.7 kcal/d, respectively (Supplemental Table 2). Mean usual intake for other macronutrients, such as protein, monounsaturated and polysaturated fatty acids, and micronutrients, such as potassium and magnesium, for tree nut consumers and nonconsumers are shown in Supplemental Table 2.

### Cardiovascular disease risk factors

Rates of hypertension (44% vs. 48%, *P =* 0.144) and diabetes mellitus (11% vs. 13%, *P =* 0.086) were not significantly different between consumers and nonconsumers. Tree nut consumers were less likely to have elevated ApoB (27% vs. 35%, *P =* 0.018) and obesity (31% vs. 40%, *P* ≤ 0.001) than nonconsumers, as shown in Table 2. Elevated ApoB and obesity prevalence were lower for tree nut consumers across race and ethnic groups (Supplemental Table 3). Tree nut consumers had a lower mean BMI (28.1 ± 0.3 vs. 29.5 ± 0.1 kg/m^2^, *P* < 0.001), SBP (121.0 ± 0.8 vs. 123.0 ± 0.2 mm Hg, *P =* 0.009), HbA1c (5.6 ± 0.03 vs. 5.7 ± 0.01 %, *P* = 0.024), and ApoB (87.5 ± 1.2 vs. 91.8 ± 0.5 mg/dL, *P =* 0.004) than nonconsumers. CVD risk factors for tree nut consumers and nonconsumers by race and ethnicity were examined and summarized in Supplement Table 3.

**TABLE 2:** Health risk factors for US adults by tree nut consumption who participated in the National Health and Nutrition Examination Survey, 2011–2018

Variables^1^	Tree Nut Consumers		NonConsumers		*P*
	≥ 7.09 g/d		< 7.09 g/d		
	(*n* = 1238)	CV	(*n* = 16912)	CV	
Cardiovascular disease risk factors					
Obesity, %	31.0 ± 2.0		40.4 ± 0.8		<.001
Hypertension, %	44.4 ±2.2		47.5 ± 0.6		0.144
TC elevated, %	32.3 ± 2.2		29.4 ± 0.6		0.188
Diabetes mellitus, %	11.0 ± 1.1		13.2 ± 0.4		0.086
Apolipoprotein B elevated, %^2^	26.6 ± 2.9		35.1 ± 0.9		0.018
BMI, kg/m^2^	28.1 ± 0.3	1.0%	29.5 ± 0.1	0.4%	<.001
SBP, mm Hg	121.0 ± 0.8	0.6%	123.0 ± 0.2	0.2%	0.009
DBP, mm Hg	70.6 ± 0.6	0.8%	71.0 ± 0.2	0.3%	0.408
TC, mg/dL	190.7 ± 1.8	1.0%	192.1 ± 0.7	0.4%	0.446
HbA_1c,_ %	5.6 ± 0.03	0.5%	5.7 ± 0.01	0.3%	0.024
ApoB, mg/dL	87.5 ± 1.2	1.4%	91.8 ± 0.5	0.6%	0.004
Cardiometabolic risk factors					
Abdominal obesity, %	54.2 ± 2.1		59.5 ± 1.0		0.014
HDL-C reduced, %	21.8 ± 1.6		29.8 ± 1.6		<.001
TG elevated, %	18.2 ± 2.1		24.3 ± 0.8		0.010
FPG elevated, %	46.7 ± 3.0		52.8 ± 0.9		0.053
WC, cm	97.1 ± 0.7	0.7%	100.5 ± 0.3	0.3%	<.001
HDL-C, mg/dL	58.6 ± 0.7	1.2%	53.3 ± 0.3	0.5%	<.001
TG, mg/dL	110.7 ± 4.7	4.2%	121.3 ± 1.7	1.4%	0.024
FPG, mg/dL	107.0 ± 1.6	1.5%	108.1 ± 0.5	0.4%	0.492

ApoB, apolipoprotein B; CV, coefficient variation; DBP, diastolic blood pressure; FPG, fasting plasma glucose; SBP, systolic blood pressure; TC, total cholesterol; WC, waist circumference.

Values are weighted means ± SE for continuous variables or weighted percentages ± SE for categorical variables.

1 Variables are defined as the following: Obesity as BMI ≥30 kg/m^2^; Hypertension as SBP ≥130 mm Hg, DBP ≥80 mm Hg, or antihypertensive medication use; elevated TC as TC ≥240 mg/dL, or lipid-lowering medication use; diabetes mellitus as FPG ≥126 mg/dL, HbA_1c_ ≥6.5% or hypoglycemic medication use; elevated ApoB ≥100 mg/dL; abdominal obesity as WC >102 cm in men and >88 cm in women; reduced HDL-C as <40 mg/dL in men and <50 mg/dL in women; elevated TG as ≥150 mg/dL; and elevated FPG as ≥100 mg/dL and/or taking antidiabetic medication.

2 Apolipoprotein B data is available only for participants in NHANES survey cycles 2011-2016.

### Cardiometabolic risk factors

Compared to nonconsumers, tree nut consumers were less likely to have abdominal obesity (54% vs. 60%, *P =* 0.014), suboptimal HDL-C (22% vs. 30%, *P* < 0.001*)*, and elevated TG (18% vs. 24%, *P =*.010) (Table 2). Reduced HDL-C and elevated TG prevalence were lower for tree nut consumers across race and ethnicity (Supplemental Table 3). Tree nut consumers had a lower mean WC (97.1 ± 0.7 vs. 100.5 ± 0.3 cm, *P* < 0.001), TG (110.7 ± 4.7 vs. 121.3 ± 1.7 gm/dL, *P =* 0.024), and a higher mean of HDL-C (58.6 ± 0.7 vs. 53.3 ± 0.3 mg/dL, *P* < 0.001), than nonconsumers. Across race and ethnic groups, tree nut consumers had higher mean HDL-C and lower mean TG (Supplemental Table 3). Cardiometabolic risk factors for tree nut consumers and nonconsumers by race and ethnicity were examined and summarized in Supplemental Table 3.

### Health outcomes

The estimated difference between average intake and low threshold intake of tree nuts was associated with BMI, HbA1c, WC, and HDL-C after adjusting for demographics and lifestyle covariates in the regression model (Model 2) though the inclusion of lifestyle covariates attenuated the associations (Table 3). The average intake was 0.49 ± 0.25 kg/m^2^ lower for BMI, 0.04 ± 0.02% lower for HbA1c, 1.42 ± 0.58 cm lower for WC, and 1.16 ± 0.43 mg/dL higher for HDL-C (Table 3) than low threshold intake of tree nuts. As shown in Table 4, when modeling the probability of the health outcomes, the usual tree nut intake was associated only with elevated FPG [Odds ratio (OR) = 0.72; 95% CI: 0.58, 0.89] after adjusting for demographics and lifestyle covariates (Model 2). The inclusion of lifestyle covariates (Model 2) attenuated the associations for all health outcomes, particularly for diabetes mellitus [OR = 0.76; 95% CI: 0.60, 0.97 (Model 1), OR = 0.80; 95% CI: 0.63, 1.02 (Model 2)] and hypertension [OR = 0.79; 95% CI: 0.63, 0.98 (Model 1), OR = 0.80; 95% CI: 0.64, 1.00 (Model 2)] (Table 4).

**TABLE 3:** Adjusted association between tree nut consumption and measures of health risk factors for US Adults who participated in the National Health and Nutrition Examination Survey, 2011–2018—Linear Regression

Outcomes – Continuous	β	SE	DIFF^1^	SE	DIFF 95% CI		*P*
					LCL	UCL	
BMI, kg/m^2^							
Model 1^2^	-0.34	0.15	-0.57	0.26	-1.08	-0.07	0.026
Model 2^3^	-0.29	0.15	-0.49	0.25	-0.97	-0.01	0.045
SBP, mm Hg							
Model 1	-0.66	0.36	-1.12	0.62	-2.33	0.09	0.069
Model 2	-0.64	0.37	-1.09	0.62	-2.30	0.12	0.077
DBP, mm Hg							
Model 1	-0.07	0.20	-0.12	0.34	-0.78	0.54	0.716
Model 2	-0.08	0.20	-0.14	0.34	-0.82	0.53	0.677
TC, mg/dL							
Model 1	-1.33	0.91	-2.25	1.53	-5.25	0.76	0.143
Model 2	-1.36	0.90	-2.31	1.52	-5.28	0.67	0.129
HbA1_c,_ %							
Model 1	-0.03	0.01	-0.06	0.02	-0.09	-0.02	0.001
Model 2	-0.03	0.01	-0.04	0.02	-0.08	-0.01	0.007
WC, cm							
Model 1	-0.99	0.37	-1.68	0.62	-2.90	-0.45	0.007
Model 2	-0.84	0.34	-1.42	0.58	-2.56	-0.28	0.015
HDL-C, mg/dL							
Model 1	0.83	0.27	1.41	0.45	0.53	2.29	0.002
Model 2	0.69	0.25	1.16	0.43	0.32	2.00	0.007
TG, mg/dL							
Model 1	-4.15	1.86	-7.02	3.15	-13.20	-0.84	0.026
Model 2	-3.43	1.84	-5.81	3.12	-11.91	0.30	0.062
FPG, mg/dL							
Model 1	-0.80	0.58	-1.35	0.98	-3.26	0.57	0.168
Model 2	-0.53	0.57	-0.89	0.96	-2.78	1.00	0.355
ApoB, mg/dL							
Model 1	-2.06	1.23	-3.48	2.08	-7.56	0.61	0.095
Model 2	-2.04	1.27	-3.46	2.15	-7.67	0.76	0.108

ApoB, apolipoprotein B; β, regression coefficient; CI, confidence interval; DBP, diastolic blood pressure; DIFF, difference; FPG, fasting plasma glucose; LCL, lower confidence limit; PIR, poverty income ratio; SBP, systolic blood pressure; TC, total cholesterol; UCL, upper confidence limit, WC, waist circumference.

1 Difference in health outcome variables between average intake (33.7 g/d, median) and low threshold intake (7.09 g/d).

2 Model 1 adjusted for age, gender, race/ethnicity, education, health insurance, PIR.

3 Model 2 adjusted for covariates of model 1 plus smoking status, physical activity.

**TABLE 4:** Adjusted association between tree nut consumption and measures of health outcomes for US adults who participated in the National Health and Nutrition Examination Survey, 2011–2018—Logistic Regression

Outcomes – Categorical^1^	OR DIFF^2^	DIFF 95% CI		
		OR LCL	OR UCL	*P*
Obesity				
Model 1^3^	0.80	0.64	1.00	0.053
Model 2^4^	0.83	0.67	1.03	0.086
Hypertension				
Model 1	0.79	0.63	0.98	0.036
Model 2	0.80	0.64	1.00	0.052
TC elevated				
Model 1	0.93	0.77	1.13	0.463
Model 2	0.95	0.79	1.15	0.609
Diabetes mellitus				
Model 1	0.76	0.60	0.97	0.029
Model 2	0.80	0.63	1.02	0.070
ApoB elevated				
Model 1	0.90	0.63	1.26	0.529
Model 2	0.90	0.63	1.29	0.577
Abdominal obesity				
Model 1	0.79	0.64	0.99	0.037
Model 2	0.82	0.66	1.01	0.058
HDL-C reduced				
Model 1	0.85	0.70	1.04	0.123
Model 2	0.90	0.74	1.09	0.273
TG elevated				
Model 1	0.82	0.63	1.07	0.143
Model 2	0.85	0.65	1.11	0.240
FPG elevated				
Model 1	0.70	0.57	0.87	0.001
Model 2	0.72	0.58	0.89	0.002

ApoB, apolipoprotein B; CI, confidence interval; DBP, diastolic blood pressure; DIFF, difference; FPG, fasting plasma glucose; OR LCL, lower confidence limit odds ratio, OR UCL, upper confidence limit for odds ratio; PIR, poverty income ratio; SBP, systolic blood pressure; TC, total cholesterol; WC, waist circumference.

1 Health outcomes defined as, obesity (BMI ≥30 kg/m^2^); hypertension (SBP ≥130 mm Hg, DBP ≥80 mm Hg, or antihypertensive medication use); elevated TC (TC ≥240 mg/dL, or lipid-lowering medication use; diabetes mellitus (FPG ≥126 mg/dL, HbA1c ≥6.5%, or hypoglycemic medication use); elevated ApoB (≥100 mg/dL); abdominal obesity (WC >102 cm in men and >88 cm in women); reduced HDL-C (<40 mg/dL in men and <50 mg/dL in women); elevated triglycerides (≥150 mg/dL); and elevated FPG (≥100 mg/dL or hypoglycemic medication use).

2 Odds ratio of health outcome difference between average intake (33.7 g/d, median) and low threshold intake (7.09 g/d).

3 Model 1 adjusted for age, gender, race/ethnicity, education, health insurance, PIR.

4 Model 2 adjusted for covariates of model 1 plus smoking status, physical activity.

As shown in Table 3, the estimated difference between average intake and low threshold intake of tree nuts was significantly associated with TG after adjustments for demographic covariates (Model 1). The average intake was 7.02 ± 3.15 mg/dL lower for TG (*P* = 0.026) than low threshold intake of tree nuts. However, the addition of lifestyle covariates (Model 2) attenuated the association, and the association was no longer statistically significant (*P* = 0.062). Other health outcomes of interest showed no statistically significant association with the usual tree nut intake. The usual tree nut intake (Model 1) was associated with lower odds for hypertension (*P* = 0.036), diabetes mellitus (*P* = 0.029), and abdominal obesity (*P* = 0.037), but the addition of lifestyle covariates (Model 2) attenuated their associations and were no longer statistically significant (*P* = 0.052, *P* = 0.070, *P* = 0.058), respectively.

## Discussion

This study showed tree nut consumption was associated with improvements in CVD and CM risk factors, albeit to a lesser extent than previously reported [18]. Yet, the results from the regression models suggest that these estimated differences between average intake and low threshold intake of tree nuts were not as profound as the mean differences suggest for tree nut consumers and nonconsumers. Demographic and lifestyle factors may attenuate the observed associations in health outcomes between average intake and low threshold intake of tree nuts.

This study found better mean BMI, WC, SBP, HDL-C, and TG among tree nut consumers consistent with other cross-sectional studies [17,18,46]. Our study does differ from previous cross-sectional analyses of the NHANES [17,18] by using more recent survey cycles and using the NCI method for modeling tree nut intake with health outcomes while accounting for the random measurement error intrinsic to the 24-h dietary recall.

A recommended nut consumption for cardiovascular prevention is 1.5 ounces per day (∼42.5 g) [[47], [48], [49]]. A concern about tree nuts is that they are high in energy and total fat, [50] yet clinical trials have demonstrated that not all energy present in tree nuts is bioavailable [[51], [52], [53], [54], [55]]. Moreover, a systematic review and meta-analysis of prospective cohorts and randomized controlled trials demonstrate that nut consumption does not lead to increased weight or measures of adiposity [56]. Our study showed a lower obesity prevalence among tree nut consumers than nonconsumers; nut consumers tended to be female, of middle to older age, with higher education and income than nonconsumers. Our study also showed a lower obesity prevalence among tree nut consumers across race and ethnic groups.

Our study used USDA FNDDS food codes rather than the US Environmental Protection Agency Food Commodity Intake Database commodity codes [18] to identify ingredients of survey foods that include nuts. The FNDDS database allows for the use of 8-digit food codes for identifying tree nuts from the study participants' dietary recalls, corresponds to the 2-y releases of the NHANES, and reports foods as having been consumed [29]. Our study also expanded on published studies using the NCI method to estimate the usual tree nut intake [18,20,57] using the SIMPLE macro wrapper with the NCI method. The SIMPLE macro facilitates reduced time and resources needed for conducting high-quality 24-h dietary analyses [42]. Furthermore, our study used 4 2-y NHANES survey cycles (2011–2018) instead of 3 2-y NHANES survey cycles (2005–2010) (18) and (1999–2004)[19]. Yet, still a relatively low percentage (∼8%) of participants consumed tree nuts, as reported in other published studies, ∼7% [18] and ∼6 % (adults 19 to 50 y) and ∼8% (adults 51+ y) [19]. Although our study sample included 1,238 tree nut consumers, equating to ∼8% of the analytic sample, the weighted number is representative of over 16.7 million individuals.

Our study used calibrated regression models for examining the relationship between usual tree nut intake and health outcomes (continuous and categorical) in adults. After adjusting for demographics and lifestyle covariates (Model 2), the usual tree nut intake was associated with BMI, HbA1c, WC, and HDL-C. Although the associations were small, our study shows how regression calibration, a further extension of the NCI method for correcting measurement error bias, can increase the precision of estimates of usual intake and of diet-health outcome associations [58]. For example, our analysis for Table 2 showed a large difference between tree nut consumers and nonconsumers for multiple mean CVD and CM risk factors, but using calibrated regression modeling demonstrated that the difference for the health outcomes was small but significant for some health outcomes, such as difference in WC (-1.42 ± 0.58 cm, *P* = 0.015) between average intake and low threshold intake of tree nuts. Precision is important since NHANES data is used to investigate the relationship between a dietary component and a disease/health outcome, which is then used to guide nutrition policy.

Our study adds to the understanding of the association between HbA1c and ApoB in place of LDL-C and tree nut consumption. Our study found that tree nut consumers had a lower prevalence of diabetes and mean HbA1c than nonconsumers, like another study that examined diabetes risk in NHANES among walnut consumers [46]. Also, our study found that tree nut consumers had a lower prevalence of elevated ApoB levels and mean ApoB than nonconsumers, as shown in Table 2. The mean ApoB was 3.46 ± 2.2 mg/dL lower for those consuming a higher (33.7 g/d) than lower (7.09 g/d) tree nut consumption level. To our knowledge, our study is the first to report the mean ApoB and elevated ApoB prevalence for tree nut consumers in a representative sample of US adults [17,18,46].

ApoB, related to LDL-C, has a role in improving the selection of individuals at risk for atherosclerotic cardiovascular disease [22]. A systematic review, meta-analysis, and dose-response of 61 controlled intervention trials found that tree nut consumption lowered ApoB, TC, LDL-C, and TG [32]. These controlled trials are useful for establishing causal links between intake and outcomes in a controlled setting. However, nationally representative samples help monitor the prevalence of selected diseases, health risk factors, or nutrients [59,60] and examine the relationship between diet and health outcomes at the population level [61,62].

Tree nuts are a good source of dietary fiber, with ranges from 4 to 11 g/100 g [63], and are part of a heart-healthy diet [64]. Tree nuts’ and peanuts’ fatty composition have been linked to beneficial effects such as cholesterol lowering [65]. Yet, nonfatty acid components of nuts, like dietary fiber, may also elicit cardioprotective effects [66]. Our study is consistent with other studies [19,20] that show tree nut consumers had a high intake of dietary fiber, along with other nutrients (potassium, magnesium, calcium, vitamin E) compared to nonconsumers. It was beyond the scope of this study to examine if the increase in dietary fiber contributed to our results, as shown in Table 3 and Table 4, which estimated the difference between average intake and low threshold intake of tree nuts.

Lastly, the 24-h recall is a valid instrument for describing intake on a consumption day and can be used with multiple administrations to approximate usual intake [67]. It is not practical in large dietary surveys as the NHANES to collect more than 2 24-h dietary recalls [68]. Thus, statistical methods like the NCI method should be employed for estimating usual dietary intake using 1 to 2 24-h dietary recalls. The NCI method addresses most measurement errors that are inherent in the analysis of 24-h data [69]. Herrick et al.[43] demonstrate in their report how using the NCI method can estimate the usual intake of nutrients consumed daily or episodically. A caveat of the NCI method is it is not a direct estimation of usual food or nutrient intake of each individual but uses “the limited data available to estimate characteristics of a group, not a single individual” [44]. Our study used the NCI method for estimating an episodically consumed food, tree nuts, which provides an analytic sample (weighted number) that is representative of over 16.7 million adult tree nut consumers.

There are several strengths of this study. Primarily, to our knowledge, it is the first study to examine the association between tree nut consumption and CVD and CM risk factors and health outcomes using calibrated regression models. Moreover, this study used a large, nationally representative sample from NHANES with proper sample weights. Furthermore, this study used standardized collection methods for exposure and outcome variables. However, we acknowledge several limitations of this study. First, the use of 24-h dietary recalls that depend on memory. Second, possible misclassification of tree nut consumers and nonconsumers due to selfreported intake. Third, limited tree nut consumption may not show health outcome improvements on the same order of magnitude as interbroad recommendation for greater intakes of tree nut consumption. Lastly, the effect of dietary fiber and total calorie intake on health outcomes was not examined.

## Conclusions

Most US adults do not consume tree nuts, yet consumption was associated with better health outcome measures. Previous analyses [18] reported that ∼6.8% of the US reported tree nut consumption, the majority of which were female (50.2%), compared with ∼8% of the respondents in the current analysis (59.9% female). Our results are consistent with previous findings that higher tree nut consumption is associated with better health outcomes, including BMI, WC, HbA1c, and HDL-C. Most CVD and CM risk factors were less severe among tree nut consumers, possibly contributing to better health. Health professionals, including registered dietitians, should advocate for the inclusion of tree nuts as part of an overall healthy dietary pattern through diet counseling and nutrition education programs. However, it is vital that health professionals are mindful of their clientele’s cultural eating patterns to mitigate challenges to the inclusion of nuts in their clientele’s daily diet [70,71]. Our study showed that usual tree nut intake was greatest among nonHispanic Blacks compared with other races and ethnicities. Yet incorporating tree nuts into the diet may be faced with additional barriers such as affordability, palatability, and allergies. Future research needs to expand the evaluation of the association between tree nut intake and health outcomes across race and ethnicity, given that health disparities vary among races and ethnic groups, and if increased consumption above observed levels is associated with improvements in cardiometabolic outcomes. Although the improvements in health outcomes associated with tree nut consumption in cross-sectional studies are less than those observed in clinical trials, they are still significant. Moreover, efforts to increase nut consumption are warranted from this and other studies, which may involve improving education, availability, affordability, and acceptance.

## Author contributions

The author’s contributions were as follows – SMLN, NZ, KSB, DCM: designed research; SMLN, DCM: conducted research; SMLN: extracted data from databases; SMLN, DCM: analyzed data; SMLN, DCM: wrote the manuscript; SMLN, NZ, KSB, DCM: reviewed and edited the manuscript; SMLN, DCM: had primary responsibility for final content; DCM: supervised the project; and all authors: read and approved the final manuscript.

## Conflict of interest

The authors report no conflicts of interest.

## Funding

The authors reported no funding received for this study.

## Data Availability

Data described in the manuscript, code book, and analytic code will be made available upon request, pending application and approval.

## Appendix A Supplementary data

Multimedia component1 Multimedia component1
