# Supplementary material for: Association of Tree Nut Consumption with Cardiovascular Disease and Cardiometabolic Risk Factors and Health Outcomes in US Adults: NHANES 2011–2018
Source: Curr Dev Nutr. 2023 Sep 25;7(10):102007. doi: 10.1016/j.cdnut.2023.102007 (PMC10585646; doi:10.1016/j.cdnut.2023.102007)
Supplement: Multimedia component1 [file mmc1.docx]

Supplementary Materials – Intended for Publication

Supplemental Tables 1, 2, 3

| **SUPPLEMENTAL TABLE 1** Usual tree nut intake among consumers in the US who participated in the National Health and Nutrition Examination Survey, 2011-2018 | | | | | | | | | | | |
| --- | --- | --- | --- | --- | --- | --- | --- | --- | --- | --- | --- |
|  |  | **Tree Nut^1^** | **Usual Intake (g/d)** | | **Percentile** | | | | | |  |
| **Study Population** |  | **Consumers (n, weighted %)** | **Mean** | **SE** | **25th** | **SE** | **Median** | **SE** | **75th** | **SE** |  |
| **Total Sample** |  | 1238, 100% | 39.5 | 1.8 | 23.0 | 1.9 | 33.7 | 1.7 | 49.4 | 2.5 |  |
| **Race/ethnicity^2^** |  |  |  |  |  |  |  |  |  |  |  |
| Mexican American | | 140, 5.9% | 33.6 | 4.4 | 20.0 | 3.4 | 29.0 | 4.1 | 41.7 | 5.4 |  |
| Other Hispanic |  | 94, 3.8% | 32.6 | 5.2 | 19.4 | 3.6 | 28.1 | 4.6 | 40.7 | 6.5 |  |
| Non-Hispanic White | | 542, 78% | 39.1 | 2.2 | 23.0 | 2.0 | 33.6 | 1.9 | 48.9 | 3.0 |  |
| Non-Hispanic Black | | 199, 6.1% | 48.2 | 3.1 | 28.7 | 2.6 | 41.8 | 2.6 | 59.7 | 4.1 |  |
| Non-Hispanic Asian | | 218, 6.3% | 35.9 | 2.5 | 21.1 | 2.3 | 30.8 | 2.4 | 44.8 | 3.2 |  |
| Estimated usual intake of tree nuts derived from using the National Cancer Institute method.  ^1^ Tree nut consumption was defined as reporting an intake of at least ¼ oz (7.09g) on a consumption day.  ^2^ Race/ethnicity groups do not sum to 100% because the "other" category is not presented per National Center for Health Statistics analytical guidelines. | | | | | | | | | | |  |
|  | | | | | | | | | | |  |

| **SUPPLEMENTAL TABLE 2** US adults’ usual nutrient intake from foods and beverages stratified by tree nut consumption: National Health and Nutrition Examination Survey, 2011-2018 | | | | | | | | |
| --- | --- | --- | --- | --- | --- | --- | --- | --- |
|  | **Tree Nut Consumers^1^** | | | | **Non-Consumers** | | | |
| **Nutrient** | n = 1311 | | | | n = 18155 | | | |
|  | **Usual Intake** | **Percentiles [SE]** | | | **Usual Intake** | **Percentiles [SE]** | | |
|  | Mean [SE] | 25^th^ | Median | 75^th^ | Mean [SE] | 25^th^ | Median | 75^th^ |
| Energy (kcal/d) | 2375 [28.7] | 2153 [27.3] | 2227 [31.2] | 2696 [31.9] | 2164 [8.7] | 1914 [9.7] | 2030 [21.8] | 2444 [13.5] |
| Protein (g/d) | 95 [1.4] | 78 [1.4] | 93 [1.5]] | 110 [1.6] | 84 [0.4] | 68 [0.6] | 83 [0.4] | 98 [0.5] |
| Carbohydrate (g/d) | 268 [4.8] | 214 [4.7] | 262 [4.9] | 316 [5.2] | 258 [1.3] | 204 [1.6] | 253 [1.3] | 306 [1.7] |
| Total fat (g/d) | 104 [1.7] | 84 [1.6] | 102 [1.7] | 121 [1.9] | 85 [0.5] | 67 [0.5] | 83 [0.5] | 101 [0.7] |
| Saturated fatty acid (g/d) | 30.5 [0.6] | 24.1 [0.5] | 29.8 [0.6] | 36.3 [0.7] | 28.1 [0.2] | 21.9 [0.2] | 27.4 [0.2] | 33.6 [0.3] |
| Monounsaturated fatty acid (g/d) | 38.7 [0.7] | 31.4 [0.6] | 37.9 [0.7] | 45.2 [0.7] | 29.8 [0.2] | 23.4 [0.2] | 29.1 [0.2] | 35.5 [0.3] |
| Polyunsaturated fatty acid (g/d) | 26.6 [0.6] | 21.6 [0.5] | 26.0 [0.6] | 31.0 [0.6] | 20.0 [0.1] | 15.7 [0.1] | 19.5 [0.1] | 23.8 [0.2] |
| Dietary fiber (g/d) | 24.4 [0.5] | 19.2 [0.5] | 23.8 [0.5] | 28.9 [0.6] | 17.2 [0.2] | 12.8 [0.1] | 16.7 [0.2] | 21.0 [0.2] |
| Potassium (mg/d) | 3333 [52.7] | 3072 [51.6] | 3254 [55.6] | 3642 [58.9] | 2682 [15.8] | 2433 [20.0] | 2662 [20.5] | 2942 [20.5] |
| Sodium (mg/d) | 3751 [53.8] | 3361 [52.3] | 3487 [58.2] | 4262 [58.4] | 3601 [16.3] | 3168 [18.6] | 3373 [37.0] | 4058 [24.9] |
| Calcium (mg/d) | 1133 [21.3] | 896 [22.4] | 1107 [22.4] | 1342 [22.3] | 985 [6.0] | 763 [8.2] | 960 [6.2] | 1181 [8.5] |
| Magnesium (mg/d) | 421.6 [6.2] | 349.8 [5.8] | 416.4 [6.4] | 487.6 [7.1] | 303.1 [1.9] | 238.9 [1.7] | 297.6 [1.9] | 361.3 [2.6] |
| Vitamin E (mg/d) | 14.0 [0.3] | 11.1 [0.3] | 13.7 [0.3] | 16.6 [0.4] | 9.3 [0.9] | 7.0 [0.06] | 9.0 [0.07] | 11.3 [0.12] |
| Estimated usual intake of nutrients derived from using the National Cancer Institute method. | | | | | | | | |
| ^1^ Tree nut consumers (≥ 7.09 g/d ) and non-consumers (<7.09 g/d). | | | | | | | | |
|  | | | | | | | | |

| **SUPPLEMENTAL TABLE 3** Risk factors for US adults by tree nut consumption by race who participated in the National Health and Nutrition Examination Survey, 2011-2018 | | | | | | | | | | | |
| --- | --- | --- | --- | --- | --- | --- | --- | --- | --- | --- | --- |
|  |  |  |  |  |  |  |  |  |  |  |  |
|  |  | **Mexican American** | | **Other Hispanic** | | **Non-Hispanic White** | | **Non-Hispanic Black** | | **Non-Hispanic Asian** | |
|  |  | Consumers^1^ | Noconsumers^1^ | Consumers | Noconsumers | Consumers | Noconsumers | Consumers | Noconsumers | Consumers | Noconsumers |
| **Variables**^2^ |  | (n = 141) | (n = 2313) | (n = 94) | (n =1810) | (n = 542) | (n = 6476) | (n = 199) | (n = 3845) | (n = 218) | (n = 1853) |
| **Cardiovascular Disease Risk Factors** | | |  |  |  |  |  |  |  |  |  |
| Obesity, % | | 45.9 ± 4.2 | 49.1 ± 1.1 | 26.8 ± 5.2 | 39.4 ± 1.5 | 29.9 ± 2.3 | 39.5 ± 1.0 | 50.6 ± 4.2 | 49.2 ± 1.0 | 10.0 ± 2.1 | 14.4 ± 0.9 |
| Hypertension, % | | 32.9 ± 5.2 | 36.7 ± 1.3 | 32.9 ± 6.6 | 38.9 ± 1.6 | 44.1 ± 2.7 | 49.0 ± 0.8 | 58.4 ± 3.1 | 56.4 ± 0.8 | 44.3 ± 3.8 | 40.8 ± 1.4 |
| TC elevated, % | | 16.9 ± 3.3 | 21.1 ± 0.9 | 20.2 ± 5.7 | 23.0 ± 1.0 | 34.4 ± 2.5 | 32.3 ± 0.6 | 28.3 ± 3.2 | 24.3 ± 0.7 | 27.1 ± 4.2 | 25.7 ± 1.2 |
| Diabetes mellitus, % | | 12.3 ± 3.0 | 15.5 ± 1.1 | 8.3 ± 3.4 | 12.7 ± 0.9 | 9.3 ± 1.3 | 12.2 ± 0.5 | 26.1 ± 3.4 | 17.0 ± 0.6 | 15.2 ± 2.5 | 14.6 ± 0.7 |
| ApoB elevated, % | | 26.1 ± 12.6 | 44.7 ± 3.2 | 41.9 ± 12.3 | 46.1 ± 2.3 | 30.5 ± 5.8 | 36.2 ± 1.7 | 34.2 ± 11.2 | 30.8 ± 2.1 | 34.0 ± 8.4 | 36.2 ± 3.1 |
| BMI, kg/m^2^ | | 30.6 ± 0.7 | 30.8 ± 0.1 | 27.6 ± 0.5 | 29.4 ± 0.2 | 27.9 ± 0.3 | 29.4 ± 0.1 | 31.3 ± 0.9 | 31.0 ± 0.2 | 25.0 ± 0.3 | 25.2 ± 0.1 |
| SBP, mm Hg | | 118.1 ± 1.5 | 120.9 ± 0.4 | 117.4 ± 2.0 | 121.2 ± 0.5 | 120.7 ± 1.0 | 123.0 ± 0.3 | 127.2 ± 1.5 | 127.1 ± 0.4 | 121.2 ± 1.4 | 120.2 ± 0.4 |
| DBP, mm Hg | | 70.3 ± 1.2 | 70.4 ± 0.3 | 69.1 ± 1.0 | 70.1 ± 0.6 | 70.4 ± 0.7 | 71.0 ± 0.3 | 73.2 ± 0.8 | 71.9 ± 0.4 | 72.1 ± 0.8 | 72.1 ± 0.3 |
| TC, mg/dL | | 186.4 ± 3.7 | 191.1 ± 1.1 | 186.0 ± 5.9 | 192.3 ± 1.3 | 191.1 ± 2.3 | 193.4 ± 0.8 | 184.6 ± 3.2 | 185.7 ± 1.0 | 190.6 ± 3.1 | 192.3 ± 1.1 |
| HbA_1c,_ % | | 5.7 ± 0.08 | 5.8 ± 0.04 | 5.6 ± 0.09 | 5.7 ± 0.03 | 5.5 ± 0.03 | 5.6 ± 0.01 | 6.1 ± 0.12 | 5.9 ± 0.01 | 5.7 ± 0.08 | 5.7 ± 0.03 |
| ApoB, mg/dL | | 87.1 ± 2.9 | 94.1 ± 1.2 | 88.3 ± 5.0 | 94.2 ± 1.2 | 87.2 ± 1.6 | 92.1 ± 0.6 | 85.4 ± 2.7 | 87.7 ± 0.9 | 92.8 ± 4.1 | 88.6 ± 1.1 |
| **Cardiometabolic Risk Factors** | | |  |  |  |  |  |  |  |  |  |
| Abdominal obesity, % | | 77.1 ± 3.6 | 69.6 ± 1.1 | 60.8 ± 6.9 | 64.7 ± 1.7 | 71.7 ± 2.8 | 72.1 ± 1.0 | 71.8 ± 3.7 | 69.8 ± 0.9 | 41.4 ± 4.6 | 40.8 ± 1.6 |
| HDL reduced, % | | 31.7 ± 4.1 | 36.7 ± 1.4 | 29.7 ± 5.6 | 35.2 ± 1.3 | 20.1 ± 1.9 | 28.8 ± 0.9 | 20.3 ± 3.2 | 25.7 ± 0.9 | 22.5 ± 3.3 | 27.8 ± 1.2 |
| TG elevated, % |  | 13.2 ± 4.4 | 29.4 ± 1.3 | 15.1 ± 6.4 | 23.8 ± 2.3 | 19.2 ± 2.7 | 25.7 ± 1.0 | 5.0 ± 2.3 | 10.9 ± 0.7 | 22.2 ± 5.6 | 25.0 ± 1.7 |
| FPG elevated, % | | 56.2 ± 5.0 | 57.0 ± 1.5 | 52.4 ± 7.2 | 49.2 ± 2.0 | 45.8 ± 4.0 | 53.3 ± 1.1 | 50.4 ± 5.2 | 47.2 ± 1.2 | 52.2 ± 5.3 | 52.0 ± 1.5 |
| WC, cm |  | 99.0 ± 1.2 | 101.8 ± 0.4 | 94.2 ± 1.3 | 98.2 ± 0.6 | 97.3 ± 0.8 | 101.2 ± 0.4 | 101.2 ± 1.9 | 101.2 ± 0.4 | 88.4 ± 0.7 | 88.9 ± 0.3 |
| HDL, mg/dL | | 52.5 ± 1.5 | 49.0 ± 0.4 | 53.9 ± 2.4 | 50.5 ± 0.4 | 59.5 ± 0.8 | 53.9 ± 0.3 | 59.7 ± 1.4 | 55.7 ± 0.3 | 56.9 ± 1.4 | 53.8 ± 0.4 |
| TG, mg/dL | | 123.5 ± 30.1 | 134.0 ± 3.5 | 105.6 ± 10.2 | 134.0 ± 8.4 | 112.0 ± 5.2 | 123.2 ± 1.9 | 75.1 ± 6.6 | 90.7 ± 2.1 | 106.4 ± 7.9 | 122.3 ± 3.5 |
| FPG, mg/dL | | 109.7 ± 3.6 | 112.0 ± 1.2 | 108.8 ± 4.4 | 109.2 ± 1.4 | 106.3 ± 1.9 | 107.5 ± 0.7 | 111.5 ± 5.5 | 108.8 ± 0.8 | 108.0 ± 2.9 | 105.8 ± 1.0 |
| ApoB, Apolipoprotein B; DBP, diastolic blood pressure; FPG, fasting plasma glucose; SBP, systolic blood pressure; TC, total cholesterol; TN, tree nuts; WC, waist circumference. | | | | | | | | | | | |
| Values are weighted means ± SE for continuous variables or weighted percentages ± SEs for categorical variables.  Estimated usual intake of tree nuts derived from using the NCI method with the SIMPLE macro wrapper. Usual tree nut intake presented in grams, means.  ^1^ Tree nut consumers (≥7.09 g/d ) and non-consumers (<7.09 g/d). | | | | | | | | | | | |
| ^2^ Variables are defined as the following: elevated ApoB ≥100 mg/dL; Obesity as BMI ≥30 kg/m2; Hypertension as SBP ≥130 mm Hg, DBP ≥80 mm Hg, or antihypertensive medication use; elevated TC as TC ≥240 mg/dL, or lipid-lowering medication use; diabetes mellitus as FPG ≥126 mg/dL, HbA_1c_ ≥6.5% or antidiabetic medication use; abdominal obesity as WC >102 cm in men and >88 cm in women; reduced HDL-C as <40 mg/dL in men and <50 mg/dL in women; elevated TG as ≥150 mg/dL; and elevated FPG as ≥100 mg/dL and/or taking antidiabetic medication. | | | | | | | | | | | |
|  |  |  |  |  |  |  |  |  |  |  |  |
